# 24 versus 48 Weeks of Peginterferon Plus Ribavirin in Hepatitis C Virus Genotype 6 Chronically Infected Patients with a Rapid Virological Response: A Non-Inferiority Randomized Controlled Trial

**DOI:** 10.1371/journal.pone.0140853

**Published:** 2015-10-28

**Authors:** Qingxian Cai, Xiaohong Zhang, Chaoshuang Lin, Xiaoqiong Shao, Yujuan Guan, Hong Deng, Min Wei, Mingshou Huang, Zefang Ren, Ling Lu, Yongyu Mei, Min Xu, Jianyun Zhu, Haiyan Shi, Guoli Lin, Ying Liu, Fengyu Hu, Qiumin Luo, Yun Lan, Fengxia Guo, Zhixin Zhao, Zhiliang Gao

**Affiliations:** 1 Department of Infectious Diseases, Third Affiliated Hospital of Sun Yat-Sen University, Guangzhou, Guangdong, China; 2 Eighth People’s Hospital of Guangzhou, Guangzhou, Guangdong, China; 3 Zhongshan Second People’s Hospital, Zhongshan, Guangdong, China; 4 Panyu People’s Hospital, Guangzhou, Guangdong, China; 5 Department of Medical Statistics and Epidemiology, School of Public Health, Sun Yat-Sen University, Guangzhou, Guangdong Province, China; 6 Laboratory for Hepatology, Third Affiliated Hospital of Sun Yat-sen University, Guangzhou, Guangdong, China; 7 Key Laboratory of Tropical Disease Control (Sun Yat-Sen University), Ministry of Education, Guangzhou, Guangdong, China; University of Athens, Medical School, GREECE

## Abstract

**Objectives:**

The optimal treatment of hepatitis C virus (HCV) genotype 6 is unclear owing to its limited geographic distribution. Because of a high predictive value of rapid virological response (RVR) for sustained virological response (SVR), we conducted an open-label randomized controlled trial to compare 24- and 48-week peginterferon/ribavirin combination therapy for patients with HCV genotype 6 in Southern China who achieved an RVR.

**Methods and Findings:**

Treatment-naive, non-cirrhotic patients with chronic hepatitis C genotype 6 were treated with pegylated interferon α-2a (180 μg/week) and ribavirin (800–1,200 mg, according to weight) for 4 weeks. Patients who achieved an RVR, which was defined as HCV RNA negativity at week 4 (<50 IU), were randomized to receive either an additional 20 or 44 weeks of treatment (24- and 48-week treatment groups, respectively). The primary outcome measure was SVR. From January 2011 to June 2014, 152(152/210, 72.4%) patients with HCV genotype 6a and RVR were randomized 1:1 to the 24- or 48-week treatment group. The SVR rates in the 24- and 48-week groups in the intention-to-treat analysis were 90.8% (69/76) and 88.2% (67/76), respectively; those in the per-protocol analysis were 95.7% (67/70) and 97.0% (64/66), respectively. More patients in the 48-week group had anemia (46.1% vs. 28.9%, *P* = 0.03), but other adverse events were comparable between the groups. The limitation of the present study was that only patients from Southern China were enrolled which may inhibit the extensive application of the findings.

**Conclusion:**

Twenty-four weeks of peginterferon/ribavirin combination therapy was non-inferior to 48 weeks in patients with HCV genotype 6a in Southern China who achieved an RVR.

**Trial Registration:**

ClinicalTrials.gov NCT01263860

## Introduction

Hepatitis C virus (HCV) is a blood-borne pathogen that infects an estimated 115 million people worldwide or approximately 1.3–2.1% of the global population [1]. HCV infection is characterized by the establishment of chronic hepatitis in approximately 70–85% of the infected individuals, among whom many develop hepatocellular carcinoma, liver cirrhosis, and liver failure, leading to liver transplantation [2, 3]. Eventually, these end-stage liver diseases cause substantial morbidity and mortality [4].

HCV was recently classified into 7 genotypes and 82 subtypes [5]. HCV subtypes 1a, 1b, 2a, 2b, and 3a are distributed globally, while all the other subtypes are largely restrictive to certain geographic regions. Genotype 6 and its subtypes are mainly found in Southeast Asia and is the most common genotype in Myanmar, Vietnam, Laos, and Cambodia [6–8]. Of the 7 genotypes, HCV genotype 6 (HCV-6) is the most diverse and includes 24 subtypes; HCV-6a is the most common subtype, accounting for 17% of HCV infections in Southeast Asia and 27% in Hong Kong [9, 10]. Studies in Southern China report that HCV-6a accounts for 49.7% of cases detected in blood donors and 51.5% of cases in intravenous drug users; furthermore, its overall proportion is increasing [11, 12].

Viral eradication is the therapeutic paradigm for chronic hepatitis C; this aims to delay liver disease progression and reduce the rates of liver failure and hepatocellular carcinoma [13]. The modern standard of care for chronic hepatitis C in Western countries is sofosbuvir-based non-interferon (IFN) combination therapy [14]. However, data on HCV-6 are scarce. In the phase III NEUTRINO trial, 6 treatment-naïve patients with HCV-6 were treated with sofosbuvir (400 mg daily) plus peginterferon (PEG-IFN) α-2a (180 μg/week) and weight-based ribavirin (RBV) (1,000–1,200 mg once daily) for 12 weeks; all achieved a sustained virological response (SVR) [15]. However, no available data support the use of a non-PEG-IFN regimen for patients with an HCV-6 infection.

Non-PEG-IFN direct-acting antiviral agents are not expected to be widely available in Asia in the near future. PEG-IFN/RBV combination therapy is still the standard of care in most Asian countries, including China. Fortunately, because of the highly favorable interleukin (IL)-28B genotype (CC genotype rs12979860 in 75.1–84.1%) [16, 17], the reported SVR rate in patients with chronic hepatitis C in Asia treated with PEG-IFN/RBV regimens (61–79%) is higher than that in Caucasians receiving PEG-IFN/RBV or triple regimens containing HCV protease inhibitors (38–41%) [18–21].

In the era of PEG-IFN/RBV, the treatment duration in patients with chronic hepatitis C is tailored according to the HCV genotype and treatment response. A rapid virological response (RVR) is the best predictor of SVR to HCV treatment [22, 23]. Furthermore, several studies have demonstrated that shorter treatment durations (i.e., 12 or 16 weeks) of PEG-IFN/RBV are as effective as a 24-week regimen for HCV-2/3 patients who have achieved an RVR [24, 25].

Among patients who were infected with HCV-1 who have lower baseline virus levels and RVR, SVR is equivalent between 24 and 48 weeks of PEG-IFN/RBV treatment [26, 27].

A recent study indicates chronic HCV-6 patients show response rates similar to those of HCV-3 patients [28]. Moreover, several studies have compared 24- and 48-week PEG-IFN treatments to chronic HCV-6. However, these studies were small and rarely involved in response-guided therapy [29, 30].

Therefore, this prospective multicenter randomized trial focused on HCV-6a, as determined by the phylogenetic analysis of HCV sequences. We hypothesized that 24 weeks of PEG-IFN/RBV treatment is sufficient for achieving an SVR rate comparable to that of the standard 48-week regimen in HCV-6a patients who have achieved an RVR.

## Methods

### Patient selection

From January 2011 to June 2014, treatment-naïve Chinese patients with chronic hepatitis C between 18 and 70 years old at four liver centers in Southern China were enrolled. The inclusion criteria were as follows: anti-HCV positivity (AMPLICOR HCV test; Roche Diagnostic Systems, Branchburg, NJ, USA) and HCV RNA positivity (COBAS AMPLICOR HCV Monitor 2.0 assay, Roche Diagnostics) (range: 50 to 69,000,000IU/mL) for >6 months; the presence of HCV-6, as determined by the phylogenetic analysis of the HCV NS5B and core fragments; elevated serum alanine aminotransferase (ALT) levels of >1.5 times the upper limit of normal; compensated liver disease (i.e., total bilirubin, <2 mg/dL; albumin, >36 g/L; prothrombin time activity >80%; and no ascites, encephalopathy, or gastrointestinal bleeding).

Exclusion criteria were the following: F4 stage liver fibrosis, defined as liver stiffness ≥12.5 kilopascal (KPa) according to the Fibroscan^®^ assessment (Echosens, Paris, France); the presence of cirrhosis or hepatocellular carcinoma, as detected on computed tomography (CT) and magnetic resonance imaging scans; HBsAg positive, or anti-human immunodeficiency virus (HIV) positive; hematological abnormalities (leukocyte count, <3,000/mL; neutrophil count, <1,500/mL; platelet count, <90,000/mL; or a hemoglobin level, <12 g/dL for women and <13 for men); alcohol consumption >20 grams per day; people who inject drugs; other liver diseases, including autoimmune liver disease and Wilson disease; receiving treatment involving any other systemic antiviral, antineoplastic, or immunomodulating drugs within 6 months prior to first dose of study drug; a history organ transplantation; or preexisting medical conditions that could interfere with their participation, including severe psychiatric illness and poorly controlled cardiac, pulmonary, or diabetic disease; or pregnancy and lactation.

### Study design

This was a randomized open-label multicenter phase III trial with active controls. All patients who met the inclusion criteria were treated with PEG-IFN α-2a (Pegasys, Roche Laboratories, Nutley, NJ, USA) 180 μg/week combined with RBV (Copegus, Roche Laboratories or Rebetol, Schering Plough) depending on body weight: 800, 1,000, and 1,200 mg/day for a body weight of ≤65, 65–75, and >75 kg, respectively[19].

RVR was defined as HCV RNA negativity at week 4 (<50 IU; COBAS AMPLICOR HCV Monitor 2.0 assay, Roche Diagnostics); patients who achieved RVR were randomized (1:1) to receive either an additional 20 or 44 weeks of combination treatment. After generating a random sequence using a random-numbers table, randomization was performed by the lead coordinator at the central site, and assignment was concealed in opaque envelopes.

Patients were followed with laboratory testing and clinical visits to assess the effiacy and safety at entry; weeks 2, 4, and 8 after the medication was administered; 4-week intervals thereafter during treatment; and at weeks 4, 12, and 24 after the end of treatment. Serum HCV RNA levels were measured at baseline, week 12, week 24, the end of treatment, and 24 weeks after the end of treatment. This study was approved by the Ethics Committee of the Third Affiliated Hospital of Sun Yat-Sen University on January 7th, 2011([2010]2–53). All patients provided written informed consent prior to participation. The study was reported according to the Consolidated Standards of Reporting Trials guidelines and was registered on ClinicalTrials.gov (ID: NCT01263860)

### Assessment of efficacy

The primary study outcome was SVR, which was defined as the absence of HCV RNA 6 months after cessation of therapy according to intention-to-treat (ITT) and per-protocol (PP) analyses. The secondary outcome measures included relapse, viral breakthrough, and dosage reduction owing to side effects (i.e., a 20% decrease in the intended dosage).

### Assessment of safety

Safety was assessed by a standardized questionnaire [31] for adverse events as well as laboratory tests performed on an outpatient basis. The World Health Organization grading system was used to grade the severity of adverse events from mild (grade 1) to life threatening (grade 4)[32]. PEG-IFN and RBV dose modifications followed the standard criteria and procedures [33].

### Statistical analysis

This was a non-inferiority trial; thus, the smallest difference considered clinically relevant was 15%. We assumed an SVR of 85% for the 48-week treatment group on the basis of results of other studies on HCV-6 [22–30]. Thus, to claim non-inferiority, the 95% confidence interval of the observed difference between the groups should not overlap >15%. With this expected SVR rate and a one-sided α of 0.05, the power was 80% for a total sample size of 138 patients with approximately 69 in each arm. Losses were estimated to be 10%. Therefore, we aimed to recruit 152 patients with RVR.

ITT and PP analyses were performed. The conclusion was conservative and was based on the analysis that detects the biggest difference.

The Student *t*-test was used to analyze continuous variables with a normal distribution, whereas nonparametric tests such as the Wilcoxon rank-sum test were used for others *χ* ^2^ statistics were used to compare categorical variables. Univariate and multiple logistic regression-stepwise backward analyses were used to calculate the adjusted odd ratios for predictors of SVR, all variables with P values of <0.25 from the univariate analysis were included in the multivariate analyses. Ninety five percent confidence intervals (95% CI) will be provided for all relevant results, the level of significance was set at P < 0.05. All statistical analyses were performed using SPSS, version 18.0 (SPSS Inc., Chicago, IL, USA.)

## Results

### Patients

**Fig 1:**
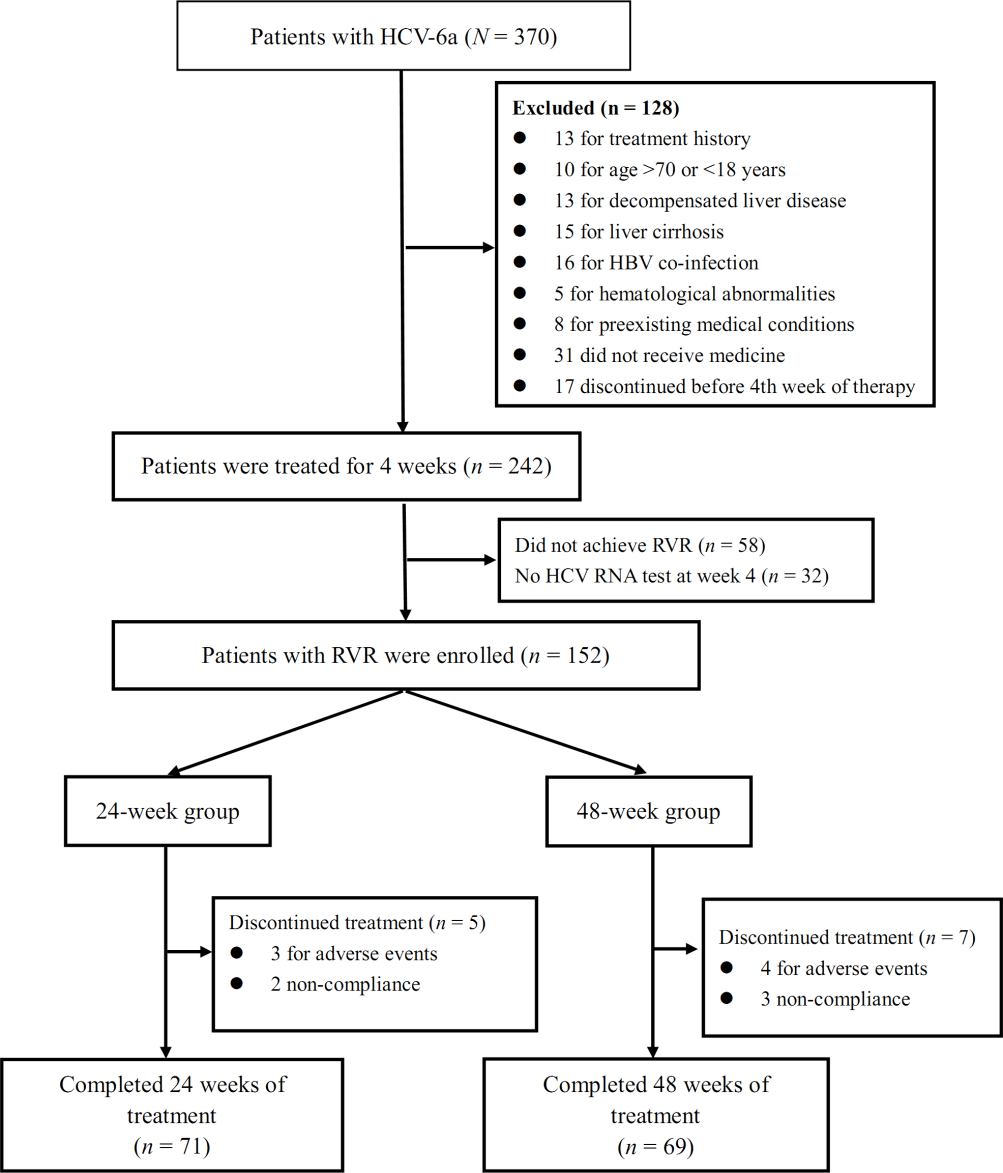
CONSORT flow diagram. Abbreviations: HBV, hepatitis B virus; HCV, hepatitis C virus; RVR, rapid virological response.

Between January 2011 to June 2014, 1,220 patients with chronic hepatitis C were successfully genotyped by the phylogenetic analysis of the NS5B and/or core regions. They were 13 (1.07%), 603 (49.43%), 156 (12.79%), 48 (3.93%), 29 (2.38%), 1 (0.08%), 366 (30.00%), 2 (0.16%), and 2 (0.16%) patients with genotypes 1a, 1b, 2a, 3a, 3b, 5a, 6a, 6e, and 6n, respectively. A flowchart of patient enrollment is shown in Fig 1. Of the 370 patients with HCV-6, 290 met the inclusion criteria; there were 179 men and 111 women with a mean age of 36.7 years (median, 36 years; range, 18–66 years). The following patients were excluded: 13 for treatment history, 10 aged >70 or <18 years, 13 for decompensated liver disease, 15 for liver cirrhosis (as determined by the Fibroscan and CT scan), 16 for hepatitis B virus co-infection, 5 for hematological abnormalities, and 8 for preexisting medical conditions. No patients had complications of liver carcinoma or HIV co-infection, and none were taking systemic antiviral, antineoplastic, or immunomodulating drugs. Of the 290 patients included, 31 did not receive a single dose of medicine, and 17 discontinued treatment before week 4 of therapy because of intolerance to the therapy.

Finally, of the 242 patients who received PEG-IFN α-2a (180 μg injected subcutaneously once per week) and weight-based RBV dosing for 4 weeks, 210 underwent HCV RNA testing at weeks 4, and 152 (152/210, 72.4%) achieved an RVR. All the 152 patients who achieved an RVR had HCV-6a and were randomized to receive an additional 20 weeks (24-week group) or 44 weeks (48-week group) of continuous combination treatment. The baseline demographic, biochemical, and virological characteristics of the patients did not differ significantly between the two groups (Table 1).

**Table 1:** Baseline patient characteristics (*n* = 152).

Characteristics	48-week group(*n* = 76)	24-week group(*n* = 76)
Age, years, mean (SD)	37.0 (10.3)	37.7 (9.7)
Age, *n* (%)		
<45 years	61 (80)	64 (84)
≥45 years	15 (20)	12 (16)
Sex, *n* (%)		
Male	46 (61)	48 (63)
Female	30 (39)	28 (37)
Route of HCV transmission, *n* (%)		
People who inject drugs	25 (33)	32 (42)
Blood or blood product transfusion	18 (24)	13 (17)
Others^a^	10 (13)	17 (22)
Unknown^b^	23 (30)	14 (18)
BMI, kg/m^2^, mean (SD)	22.9 (2.8)	23.3 (3.4)
BMI, *n* (%)		
<24 kg/m^2^	55 (72)	45 (59)
≥24 kg/m^2^	21 (28)	31 (41)
Serum ALT, *n* (%)		
<3 UNL^c^	58 (76)	65 (86)
≥3 UNL	18 (24)	11 (14)
Fibroscan score		
F0/F1 (0–7.3)	54 (71)	58 (76)
F2/F3 (7.4–12.4)	22 (29)	18 (24)
Serum HCV RNA, log10IU/mL, mean (SD)	6.3 (1.0)	6.4 (0.8)
<400,000 UI/mL, *n* (%)	33 (43)	25 (33)
≥400,000 UI/mL, *n* (%)	43 (57)	51 (67)
IL-28B 12979860		
CC	65 (86)	70 (92)
CT/TT^d^	11 (14)	6 (8)

Abbreviations: ALT, alanine aminotransferase; UNL, upper limit of normal; BMI, body mass index; HCV, hepatitis C virus; IL, interleukin

^a^ Surgery, intravenous injection, dentist visit, hemodialysis, tattooing, cosmetology, occupational exposure, or intercourse with an HCV-infected person

^b^ The patient did not recall any specific exposure risk.

^c^ Patients were attributed into two groups according to ALT lower and not lower than 3 times of upper limit of normal.

^d^ No patient had the IL-28B rs12979860 TT genotype.

Overall, 71 and 69 patients in the 24- and 48-week groups, respectively, completed the planned treatment. Patients were asked to finish the 24-week post-treatment follow-up regardless of whether they completed or discontinued treatment. Finally, 73 and 70 patients in the 24- and 48-week groups, respectively, reached their primary endpoint evaluation 24 weeks after the termination of therapy.

### Treatment response

Overall, 71 and 73 patients in the 24- and 48-week groups, respectively, underwent HCV RNA PCR testing at week 12 of treatment, and all achieved a complete and early virological response. All the patients had undetectable HCV RNA levels at the end of treatment, and no virological breakthrough was detected during treatment.

For ITT analysis, the primary endpoint was analyzed on the basis of 76 patients enrolled in each group. Only those who had undetectable HCV RNA levels at week 24 after treatment cessation were treated as having achieved an SVR. The SVR rates in the 24- and 48-week groups were 90.8% (69/76) and 88.2% (67/76), respectively; there was no significant difference between the groups (P = 0.597). The relapse rates in the 24- and 48-week groups were 5.3% (4/76) and 3.9% (3/76), respectively (*P* = 0.70) (Table 2).

**Table 2:** Treatment adherence and primary endpoint analysis at the end of the follow-up.

	Treatment adherence		PP analysis		PP analysis		
	Completed	Discontinued	*n*	SVR (*n*, %)	*n*	SVR (*n*, %)^a^	Relapse (*n*, %)^a^
24-week	71	5	70	67/70, 95.7	76	69/76, 90.8	4/76, 5.3
48-week	69	7	66	64/66, 97.0	76	67/76, 88.2	3/76, 3.9

Abbreviations: PP, per protocol; SVR, sustained virological response

^a^ SVR rate in the per-protocol analysis

For PP analysis, the primary endpoint was analyzed in the 70 and 66 patients in the 24- and 48-week groups, respectively, who completed the planned treatment duration and follow-up. The SVR rates in the 24- and 48-week groups were 95.7% (67/70) and 97.0% (64/66), respectively (*P* = 0.697) (Table 2).

### Predictors of SVR

The factors associated with SVR were analyzed among all patients who completed treatment and follow-up (in the PP analysis). However, the HCV RNA level, age, sex, route of HCV transmission (i.e., people who inject drugs vs. people who do not inject drugs), body mass index (BMI) (i.e., ≥24 kg/m^2^ vs. <24 kg/m^2^), serum ALT (i.e., ≥3 UNL vs. <3 UNL), fibroscan score (i.e., ≥7.4 KPa vs. <7.4 KPa), and IL-28B rs12979860 genotype (i.e., CC vs. non-CC) were not significant predictors of SVR in univariate or multivariate analysis (all, *P* > 0.05)

We also compared SVR in a subgroup analysis according to two major predictors: the HCV RNA level (i.e., < or ≥400,000 IU/mL [low and high viremia, respectively]) and IL-28B rs12979860 genotype (i.e., CC vs. non-CC genotype). In patients in the 24-week group, the SVR rates of those with low and high viremia were 100% (23/23) and 93.6% (44/47), respectively; those in the 48-week group were 96.7% (29/30) and 97.2% (35/36), respectively, which are comparable. Meanwhile, the SVR rates in patients in the 24-week group with rs12979860 CC and non-CC genotypes were 96.9% (62/64) and 83.3% (5/6), respectively; those in the 48-week group were 96.5% (55/57) and 100% (9/9), which are comparable.

### Safety and adherence

All the enrolled patients were included in the safety analysis. The most common adverse events were influenza-like symptoms. The incidence of most treatment-related adverse events was similar in both treatment groups (Table 3). Significantly more patients (46.1%) in the 48-week group had mild anemia (hemoglobin <11 g/dL) than patients (28.9%) in the 24-week group (mean difference -1.3%, 95% CI -7.6% to 5.0%). Moreover, 18 (23.7%) and 27 (35.5%) patients in the 24- and 48-week groups had hemoglobin levels <10 g/dL and thus required RBV reduction (P = 0.11), and 15 patients (19.7%) in the 24-week groups and 24 patients (31.6%) in 48-week groups (P = 0.09) received subcutaneous injections of erythropoietin. In the 24- and 48-week groups, 7 (9.2%) and 10 (13.2%) patients, respectively, had neutrophil counts <750/μL and thus required IFN dose reduction and granulocyte colony-stimulating factor administration (*P* = 0.44).

**Table 3:** Treatment-related adverse events.

Adverse event, *n* (%)	24-week (*n* = 76)	48-week (*n* = 76)	*P*-value
Asthenia	48 (63.2)	41 (53.9)	0.25
Influenza-like symptoms^a^	61 (80.3)	56 (73.7)	0.34
Gastrointestinal symptoms^b^	49 (64.5)	54 (71.1)	0.39
Dermatologic symptoms^c^	17 (22.4)	27 (35.5)	0.07
Psychiatric symptoms^d^	13 (23.2)	21 (29.1)	0.45
Hematological abnormalities	43 (56.6)	54 (71.1)	0.06
Anemia			
Hb < 11 g/dL	22 (28.9)	35 (46.1)	0.03
Hb < 10 g/dL	18 (23.7)	27 (35.5)	0.11
Hb < 8.5 g/dL	4 (5.3)	7 (7.9)	0.51
Neutropenia			
ANC < 1500/μL	33 (43.4)	41 (53.9)	0.19
ANC < 750/μL	7 (9.2)	10 (13.2)	0.44
ANC < 500/μL	4 (5.3)	3 (3.9)	0.7
Thrombocytopenia			
Platelet count < 90/μL	12 (15.8)	14 (18.4)	0.67
Platelet count < 50/μL	1 (1.3)	3 (3.9)	0.31
Platelet count < 25/μL	0 (0)	1 (1.3)	0.32
Thyroid disease	2 (2.6)	3 (3.9)	0.65
Diabetes mellitus	0 (0)	1 (1.3)	0.32

Abbreviations: Hb, hemoglobin; ANC, absolute neutrophil count

^a^ Influenza-like symptoms: pyrexia, rigors, headache, and myalgia

^b^ Gastrointestinal symptoms: anorexia, nausea, and vomiting

^c^ Dermatologic symptoms: dermatitis, pruritus, and alopecia

^d^ Psychiatric symptoms: insomnia, depression, and irritability

In the 24- and 48-week groups, 2 (2.6%) and 3 (3.9%) patients, respectively, had platelet counts <50/μL and thus required IFN dose reduction and megakaryocyte colony-stimulating factor administration (*P* = 0.31). Overall, 18 (23.7%) and 22 (28.9%) patients in the 24- and 48-week groups (*P* = 0.46), respectively, required dose reduction.

Treatment was interrupted or discontinued when mild anemia (hemoglobin <8.5 g/dL), grade 4 neutropenia (absolute neutrophil count, <500/μL), or thrombocytopenia (platelet count, <25/μL) occurred. Therapy was permanently discontinued in 5 patients of the 24-week group, including 3 for adverse events and 2 for non-compliance, and in 7 patients of the 48-week group, including 4 for adverse events and 3 for non-compliance (Table 2).

## Discussion

In this investigator-initiated open-label randomized controlled trial that evaluated the role of RVR for determining the treatment duration for HCV-6 patients, the ITT and PP analyses demonstrated that 24 weeks of PEG-IFN/RBV combination therapy is non-inferior to 48 weeks of therapy when an RVR is achieved. RVR, which was defined as undetectable HCV RNA (<50 IU/mL) at week 4 of PEG-IFN/RBV therapy, was achieved in 72.4% of patients who were infected with HCV-6. Moreover, HCV-6 patients who achieved RVR also achieved a high SVR rate of >88% in the ITT analysis, which was even higher (>95%) in the per-protocol analysis.

Approximately 24–27% and 64–86% of patients with HCV-1 and HCV-2/3 achieved an RVR, respectively [18, 24, 33]; moreover, 42–46% and 76–82% achieved an SVR, respectively [34, 35]. The outcomes of HCV-6 patients treated with PEG-IFN/RBV were superior to those of HCV-1 patients [28, 36] and were comparable to those of HCV-2/3 patients; the RVR and SVR rates of HCV-6 patients were >70% and 60–90%, respectively [29, 30, 37]. The findings from the present study corroborate these findings.

HCV-6 is confined to Southeast Asia, Hong Kong, and Southern China where people tend to have a lower BMI, consume less alcohol, and have more favorable IL-28B genotypes than people from Western countries do. Most patients in the present study had a BMI <24 kg/m^2^ and had an rs12979860 CC genotype. These factors may contribute to the high RVR and SVR rates of the HCV-6 patients in these regions [3, 16, 17]. However, it remains unclear whether the high SVR in the HCV-6 patients is due to viral factors alone or host factors as well.

The optimal treatment duration of HCV-6 patients remains controversial. A retrospective cohort study by Nguyen et al. [38] showed that 48-week PEG-IFN/RBV treatment results were associated with a significantly higher SVR rate than 24-week treatment (75% vs. 39%). In contrast, in a randomized controlled study by Lam et al. [29], 60 HCV-6 patients were randomized to 24- or 48-week treatment, but the SVR rates were comparable (79% vs. 70%); however, the RVR rate in the 24-week treatment group tended to be higher than that in the 48-week group (17/20 vs. 12/19), which may significantly narrow the difference in the SVR rate between the groups. In a randomized controlled study published in 2012, 105 HCV-6 patients were randomized to 48-week or 24-week PEG-IFN/RBV treatment; there was no significant difference in SVR between the groups (71% vs. 60%); that study performed post hoc subgroup comparisons of the SVR rates in patients who achieved RVR but found no significant difference [37]. However, because of the superiority trial design and the small sample of RVR patients involved, the study did not claim the non-inferiority of 24-week PEG-IFN α-2a/RBV treatment compared to 48-week treatment.

The present study was designed on the basis of the non-inferiority principle. We published ITT analysis findings, because this is the standard methodology for presenting data in a randomized controlled trial. However, for a non-inferiority study, PP analysis provides more stringent and empirical results. Therefore, the present study claimed that 24-week PEG-IFN/RBV treatment is non-inferior to 48-week treatment for HCV-6 patients who achieve an RVR.

Previous studies have shown that the treatment outcomes of chronic hepatitis C are associated with the genotype, HCV RNA viral load, age, sex, BMI, metabolic syndrome, insulin resistance, alcohol consumption, and liver disease characteristics, including ALT and gamma-glutamyl transferase levels, fibrosis stage, and co-infection with another hepatotropic virus or HIV [22, 29, 30, 39].

Besides the HCV genotype, one of the most robust baseline predictors of SVR is the baseline HCV RNA load. A recent randomized trial by Yu et al. demonstrated that in HCV-1 patients with low viremia and RVR, 24- and 48-week treatments resulted in similar SVR rates, but 24-week PEG-IFN/RBV treatment was inferior to 48-week treatment in patients with high viremia, even if they achieved an RVR. In patients with HCV-2/3, shorter PEG-IFN/RBV treatment durations from 12–16 weeks are as effective as a 24-week regimen when an RVR is achieved [24, 25, 33]. However, a recent large trial on HCV-2/3 patients showed that 24 weeks is superior to 16 weeks in patients with high viremia [34]. Moreover, because different studies choose different cut-offs as high or low [18,33,40], it is very difficult to define the exact level at which a patients’ likelihood of SVR will be diminished, which makes it difficult to risk-stratify or prognosticate the patient’s chances for achieving SVR based on the level of viremia in clinical practice. Until recently, the predictive value of baseline viremia for HCV-6 patients treated with PEG-IFN/RBV has not been clarified in any large clinical studies.

In this study, the SVR rate was equivalent with 24 and 48 weeks of treatment, even in subgroups stratified according to the HCV RNA level. This indicates that RVR can serve as a guide for tailoring the treatment duration of PEG-IFN/RBV for HCV-6.

IL-28B polymorphisms are associated with SVR and RVR to PEG-IFN/RBV treatment in HCV-1 [41–44] and HCV-2/3 [45, 46]. However, IL-28B genomic-based treatment paradigms for chronic hepatitis C infection need to be demonstrated in clinical trials. Owing to the geographical distribution of HCV-6, data on the predictive value of IL-28B in HCV-6 are scarce. In this study, the SVR rate did not differ with respect to the IL-28B rs12979860 genotype (CC vs. non-CC). This finding implies that RVR itself can objectively help to determine whether a patient should receive a 24- or 48-week course of PEG-IFN/RBV treatment. This would also minimize the need to perform IL28 genotyping in all patients, which is more likely to be more costly than monitoring RVR to help guide treatment.

As the distribution of the CC IL-28B genotype may be more common in Asian patients than the CT and TT genotypes, the predictive value of IL-28B for HCV-6 needs to be demonstrated in a larger trial.

The most common adverse events were general non-specific symptoms and hematological abnormalities; all were mild and manageable with supportive measures. A few required treatment dose reduction or interruption until the blood test results returned to normal. Only a few patients with severe adverse events were discontinued permanently. As reported in previous HCV treatment trials, the incidence and types of adverse events due to PEG-IFN/RBV therapy appear to be similar among patients with different HCV genotypes, but they differ with respect to ethnicity [47, 48]. Hu et al. reported that compared to Caucasian and Hispanic patients, Asian patients tend to have anemia more frequently and fewer psychiatric adverse events [49]. Subsequent studies reported no significant differences between Caucasians and Asians with respect to the required RBV or PEG-IFN dose reductions [50, 51]. Because of the geographic distribution of HCV-6, the adverse event profiles in the present study were quite similar to those of clinical trials from Asia but were different from those in Western studies [34, 35, 52]. In this study, mild anemia was more common in the 48-week group, which is probably due to the longer exposure to RBV. However, no difference was found between groups with respect to non-specific symptoms, anemia (<10 g/dL), severe anemia (<8.5 g/dL), treatment discontinuation, or dose reduction. This may be because most adverse events occur in the early treatment phase. Shortening the treatment duration did not substantially decrease the prevalence of adverse events. Nevertheless, shortening the suffering of adverse effect would substantially improve patients’ quality of life.

Generally, the present study adds to our knowledge of patients with hepatitis C and genotype 6 which is the most common genotype in Southeast Asia where disease burden is very high, but has been a relatively neglected area in the literature. Currently, PEG-IFN/RBV combination therapy is still the standard of care in most Asian countries, including China. The findings in the present study will greatly help to optimize the treatment regimen for HCV-6a patients in Asian countries where non-PEG-IFN direct-acting antiviral agents are not expected to be widely available in the near future.

One limitation of the present study was that only patients from Southern China were enrolled; thus, a diverse culture and lifestyle from a different country might influence the study findings. Furthermore, patients in the current study were much younger and had less severe liver fibrosis than patients from other clinical trials did [30, 38]. These differences in the patients’ characteristics may account for the high SVR observed in the current study, because older age and more severe liver fibrosis are associated with a lower likelihood of achieving SVR. Moreover, 31 patients withdrew after enrollment with unknown reasons. Thirty two patients had no HCVRNA result at week 4 of treatment. In conclusion, 24-week PEG-IFN/RBV treatment was non-inferior to 48-week treatment for HCV-6 patients in Southern China who achieved an RVR.

## Supporting Information

S1 CONSORT Checklist CONSORT Checklist. (DOCX)

S1 Fig Primary ethical approval. (PDF)

S1 File Dataset .
(XLSX)

S1 Protocol Protocol in English. (DOCX)

S2 Protocol Protocol in Chinese.doc. (DOC)

S1 Text Translation of ethical approval. (PDF)

## Abbreviations

ALT: alanine aminotransferase
BMI: body mass index
HCV: hepatitis C virus
ITT: intention to treat
KPa: kilopascal
PEG-IFN: peginterferon
PP: per protocol
RBV: ribavirin
RVR: rapid virological response
SVR: sustained virological response
