# Supplementary material for: 24 versus 48 Weeks of Peginterferon Plus Ribavirin in Hepatitis C Virus Genotype 6 Chronically Infected Patients with a Rapid Virological Response: A Non-Inferiority Randomized Controlled Trial
Source: PLoS One. 2015 Oct 28;10(10):e0140853. doi: 10.1371/journal.pone.0140853 (PMC4624894; doi:10.1371/journal.pone.0140853)
Supplement: S1 Fig — (PDF) [file pone.0140853.s002.pdf]

中山大学附属第三医院医学伦理委员会临床研究审批件

编号：中大附三医伦[2010]2-53 号

项目名称	基因 6 型慢性丙型肝炎的个体化优化治疗及长期预后的前瞻性研究		
专业科室	感染科	项目负责人	赵志新
研究课题来源	申报中山大学临床医学研究 5010 计划项目		
研究经费来源	申报中山大学临床医学研究 5010 计划项目资助		

已审查文件：

1. 研究方案
2. 知情同意书

医学伦理委员会审查结论如下：

☑批准

☐修改后再审查

☐不批准

主任委员/副主任委员：[illegible]

2011 年 1 月 7 日

注意事项：

1. 本伦理委员会的职责、人员组成、工作程序遵循卫生部《涉及人的生物医学研究伦理审查办法》等国家的相关法律法规和规章。
2. 研究如获中山大学临床医学研究 5010 计划项目批准立项，请报伦理委员会备案。
3. 研究过程中，对研究方案和知情同意书等相关文件所作的任何修改，均须得到伦理委员会审查同意后方可实施。
4. 在实施过程中发生严重不良事件的，应当及时向伦理委员会报告。
5. 研究结束后请向伦理委员会提交总结报告。

中山大学附属第三医院医学伦理委员会
通讯地址：广东省广州市天河路 600 号　　邮编：510630　　Tel（Fax）：85253099
