# Supplementary material for: 24 versus 48 Weeks of Peginterferon Plus Ribavirin in Hepatitis C Virus Genotype 6 Chronically Infected Patients with a Rapid Virological Response: A Non-Inferiority Randomized Controlled Trial
Source: PLoS One. 2015 Oct 28;10(10):e0140853. doi: 10.1371/journal.pone.0140853 (PMC4624894; doi:10.1371/journal.pone.0140853)
Supplement: S1 Protocol — (DOCX) [file pone.0140853.s004.docx]

**A randomized trial of 24-week versus 48-week courses of peginterferon plus ribavirin for hepatitis C virus genotype 6 chronically infected patients with rapid virological response**

ClinicalTrials.gov:  NCT01263860

**Financial support:** This study was supported by funding from the National Science and Technology Major Project (2012ZX10002003) and Sun Yat-Sen University Clinical Research 5010 Program (2010011).

**Roles and responsibilities**

Zhiliang Gao, Doctor

Principal Investigator

Department of Infectious Diseases, Third Affiliated Hospital of Sun Yat-Sen University, No 600, Tianhe Road, Tianhe, Guangzhou, China

Tel: 8620-85252373; Email: [Zhilianggao@21cn.com](mailto:Zhilianggao@21cn.com)

Zhixin Zhao, Doctor

Principal Investigator

Department of Infectious Diseases, Third Affiliated Hospital of Sun Yat-Sen University

No 600, Tianhe Road, Tianhe, Guangzhou, China Guangdong, China, 510630

Tel: 8620-85252372; Email: [cqx200000@163.com](mailto:cqx200000@163.com)

Yujuan Guan, Doctor

Study Principal Investigator

Eighth People’s Hospital of Guangzhou, Guangzhou, Guangdong, China

627 East Dongfeng Road, Guangzhou 510060, China

Email: guanyj9992@163.com

Wei min, Doctor

Study Principal Investigator

Zhongshan second people's hospital

Email: doctor.Wm@163.com

Huang mingshou, Bachelor

Study Principal Investigator

Panyu People's Hospital

Email:hms3388@126.com

**Trial Sponsor:** Sun Yat-Sen University

**1 Background**

Hepatitis C virus (HCV) infection is a major cause of cirrhosis and hepatocellular carcinoma, and often requires liver transplantation. Approximately 3% of the global population is chronically infected with HCV (1, 2). In the Asia-Pacific region, the prevalence of HCV infection ranges from 2.2% in Southeast Asia to 3.9% in the Western Pacific (3).

HCV was recently classified into 6 genotypes: HCV genotypes 1–3 are distributed globally; genotypes 4 and 5 are found mainly in Africa and the Middle East; and genotype 6 and its subtypes are found mainly in Southeast Asia and is the most common genotype in Myanmar, Vietnam, Lao, and Cambodia (4-6). HCV-6a is the most common subtype, accounting for 17% of HCV infections in Southeast Asia and 27% in Hong Kong (7, 8). Studies in Southern China report that HCV-6a accounts for 49.7% of cases detected in blood donors and 51.5% of cases in intravenous drug users; furthermore, its overall proportion is increasing (9, 10).

In the era of PEG-IFN/RBV, treatment duration in patients with chronic hepatitis C is tailored according to HCV genotype and treatment response. Rapid virological response (RVR) is the best predictor of SVR to HCV treatment (11, 12). Furthermore, several studies demonstrate shorter treatment duration (i.e., 12 or 16 weeks) of PEG-IFN/RBV is as effective as a 24-week regimen for HCV-2/3 patients who have archived an RVR (13, 14).

A recent study indicates chronic HCV-6 patients show response rates similar to those of HCV-3 patients (15). However, the treatment is associated with many and sometimes serious side effects. In addition, the treatment is costly also in economic terms. Shorter treatment for chronic hepatitis C genotype 6 is necessary to be assessed.

**2 Objectives**We hypothesized 24-week of PEG-IFN/RBV treatment is sufﬁcient to achieve an SVR rate comparable to that of the standard 48-week regimen in HCV-6a patients who have achieved an RVR.

**2.1 Primary objective**

To determine if 24-week of PEG-IFN/RBV treatment is noninferior to 48-week regimen in HCV-6a patients who have achieved an RVR.

**2.2 Secondary objectives**

To compare the adverse events and sick leave between patients treated with 24-week and 48-week of PEG-IFN/RBV treatment

**3 Study design**

The study is designed as a randomized open-label multicenter non-inferiority trial with with an active control and a primary endpoint of sustained virological response. Randomization will be performed as simple randomization with a 1:1 allocation.

**3.1 Study setting**

Studies in Southern China report that HCV-6a accounts for 49.7% of cases detected in blood donors and 51.5% of cases in intravenous drug users. The study were decided to be carried out in the following four centers: Third Affiliated Hospital of Sun Yat-Sen University, Eighth People’s Hospital of Guangzhou, Zhongshan second people's hospital and Panyu People's Hospital.

**3.2 Inclusion Criteria**

- Chinese patients from the four liver centers;
- Anti-HCV positivity and HCV RNA positivity for >6 months;
- Interferon treatment-naïve;
- Age from 18 to 70 years;
- HCV genotype 6 determined by phylogenetic analysis of the HCV NS5B and Core fragments;
- Compensated liver disease (i.e., total bilirubin <2 mg/dL; albumin >36 g/L; prothrombin time activity >80%; and no ascites, encephalopathy, or gastrointestinal bleeding);

**3.3 Exclusion criteria included:**

- Liver stiffness ≥12.5 kilopascal (KPa) according to the assessment by Fibroscan® (Echosens, Paris, France);
- Cirrhosis or hepatocellular carcinoma detected by CT or MRI scans;
- HBsAg positive, or anti-human immunodeficiency virus (HIV) positive;
- Hematological abnormalities (leukocyte count <3,000/mL, neutrophil count <1,500/mL, platelet count <90,000/mL, or a hemoglobin <12 g/dL for women and <13 for men);
- Alcohol consumption greater than 20 grams daily;
- The presence of drug abuse;
- Other liver diseases, including autoimmune liver disease and Wilson disease;
- Receiving treatment involving any other systemic antiviral, antineoplastic, or immunomodulating drugs within 6 months prior to ﬁrst dose of study drug;
- A history organ transplantation;
- Preexisting medical conditions that could interfere with their participation, including severe psychiatric illness and poorly controlled cardiac, pulmonary, or diabetic disease;
- Pregnancy or lactation.

**3.4 Intervention**

All patients who met the inclusion criteria were treated with PEG-IFN α-2a (Pegasys, Roche Laboratories, Nutley, NJ, USA) 180 μg/week combined with RBV (Copegus, Roche Laboratories or Rebetol, Schering Plough) depending on body weight: 800, 1,000, and 1,200 mg/day for a body weight of ≤65, 65–75, and >75 kg, respectively.

RVR was defined as HCV RNA negativity at week 4 (<50 IU; COBAS AMPLICOR HCV Monitor 2.0 assay, Roche Diagnostics); Patients who achieved RVR were randomized (1:1) to receive either an additional 20 or 44 weeks of combination treatment.

**3.5 Follow up**

Patients were followed up by laboratory testing and clinical visits to assess efﬁcacy and safety at entry; treatment weeks 2, 4, and 8; 4-week intervals thereafter during treatment; and at weeks 4, 12, and 24 after the end of treatment. Serum HCV RNA levels were measured at baseline, week 12, week 24, the end of treatment, and 24 weeks after the end of treatment. This study was approved by the Ethics Committee of the Third Affiliated Hospital of Sun Yat-Sen University. All patients provided written informed consent prior to participation.

**4 Primary Outcome Measures**

- **Sustained virological response (SVR)：**Undetectable HCV RNA (<50 IU/mL) 24 weeks after the end of therapy;

**5 Secondary Outcome Measures**

- **Complete EVR:** Non-detectability of HCV RNA (<50 IU/mL) after 12 weeks of therapy
- **Partial EVR:** At least a 2 log10 decrease in HCV RNA (IU/mL) from baseline level after 12 weeks of therapy;
- **Rapid virological response:** Non-detectability of HCV RNA (\50 IU/mL) after 4 weeks of therapy;
- **End-of-treatment (virological):** Non-detectability of HCV RNA at the end of therapy;
- **Response:** Undetectable HCV RNA at the end of therapy, but reappearance of HCV RNA after the end of therapy;
- **Breakthrough:** Reappearance of HCV RNA at any point during treatment after virological response;
- **Partial response or partial nonresponse:** More than 2 log10 decrease in HCV RNA (IU/mL) from baseline at 12 weeks of therapy, but detectable HCV RNA at week 24.

**6 Sample size**

The sample size was calculated on the basis of the primary hypothesis. This was a non-inferiority trial; thus, the smallest difference considered to be clinically relevant was 15%. We assumed an SVR of 85% for the 48-week treatment group on the basis of results of other studies on HCV-6 (11-17). Thus, to claim “non-inferiority,” the 95% confidence interval of the observed difference between the groups should not overlap >15%. With this expected SVR rate and a one-sided α of 0.05, the power was 80% for a total sample size of 138 patients with approximately 69 in each arm. Losses were estimated to be 10%. Therefore, we aimed to recruit 152 patients with RVR.

ITT and PP analyses were performed. The conclusion was conservative and based on the analysis that detects the biggest difference.

**7 Randomization and allocation**

All patients who give consent for participation and who fulfil the inclusion criteria will be randomized. Randomization will be requested by the lead coordinator at the central site from the statistician. The statistician will send an answer form enclosed a randomization number to the study therapist who is not going to assess outcome of the study. Each center will have a closed opaque envelopes with printed randomization which indicate the corresponding code for the therapy group. The therapist are pointed to open the envelope to assign the patient to the treatment group.

To ensure the Allocation concealment, staff responsible for recruitment and symptom ratings are not allowed to receive information about the group allocation.

**8 Data collection**

All data will be entered electronically. Original case report forms will be entered and kept on file at the study center. But for quality control, the participating center staff may have to copy a form selected and sent the copies to data coordinating center for re-entry.

Participant files will be stored in a secure and accessible place. All the files will be storage for 5 years after completion of the study.
**9 Access to data**

All Principal Investigators will be given access to the study data set, and all data sets will be protected with passwords. All of the Principal Investigators will possess direct access to their own site’s data sets, but could not access to other sites data unless their request was permitted. For confidentiality, any identifying participant information would not appear in the data dispersed to project team members.

**10 Statistical methods**Two study groups will be compared for all primary analysis. Chi-squared test will be used for binary outcomes. Student’s *t*-test was used to analyze continuous variables with a normal distribution, whereas nonparametric tests such as the Wilcoxon rank-sum test were used for others. *χ*^2^ statistics were used to compare categorical variables. Both ITT and PP analyses were performed. The conclusion was conservative and based on the analysis that detects the biggest difference. Univariate and multiple logistic regression-stepwise backward analyses were used to calculate the adjusted odd ratios for predictors of SVR. We will calculate Relative Risk (RR) and RR Reductions (RRR) with corresponding 95% confidence intervals to compare dichotomous variables, and difference in means will be used for additional analysis of continuous variables. The level of significance was set at P < 0.05 (one-sided). All statistical analyses were performed using SPSS, version 18.0 (SPSS Inc., Chicago, IL, USA.)

**Reference**

1. Lauer GM, Walker BD. Hepatitis C virus infection. N Engl J Med 2001; 345:41–52.
2. [Lavanchy D](http://www.ncbi.nlm.nih.gov/pubmed/?term=Lavanchy%20D%5BAuthor%5D&cauthor=true&cauthor_uid=19207969). The global burden of hepatitis C. Liver Int 2009; 29:74-81
3. McCaughan GW, Omata M, Amarapurkar D, et al. Asian Pacific Association for the Study of the Liver (APASL) Hepatitis C Working Party.Asian Pacific Association for the Study of the Liver consensus statements on the diagnosis, management and treatment of hepatitis C virus infection. J Gastroenterol Hepatol 2007; 22:615-633.
4. Pham DA, Leuangwutiwong P, Jittmittraphap A, et al. High prevalence of Hepatitis C virus genotype 6 in Vietnam. Asian Pac J Allergy Immunol 2009; 27:153-60.
5. Hübschen JM, Jutavijittum P, Thammavong T, et al. High genetic diversity including potential new subtypes of hepatitis C virus genotype 6 in Lao People's Democratic Republic. Clin Microbiol Infect. 2011; 17:E30-34.
6. Akkarathamrongsin S, Praianantathavorn K, Hacharoen Nm, et al.Seroprevalence and genotype of hepatitis C virus among immigrant workers from Cambodia and Myanmar in Thailand. Intervirology 2011; 54:10-16
7. Dev AT, McCaw R, Sundararajan V, et al. Southeast Asian patients with chronic hepatitis C: the impact of novel genotypes and race on treatment outcome. Hepatology 2002; 36:1259-1265
8. Prescott LE, Simmonds P, Lai CL, et al. Detection and clinical features of hepatitis C virus type 6 infections in blood donors from Hong Kong.J Med Virol 1996; 50:168-175..
9. Fu Y, Wang Y, Xia W, et al. New trends of HCV infection in China revealed by genetic analysis of viral sequences determined from first-time volunteer blood donors. J Viral Hepat 2011; 18:42-52.
10. Fu Y, Qin W, Cao H, et al. HCV 6a prevalence in Guangdong province had the origin from Vietnam and recent dissemination to other regions of China: phylogeographic analyses. PLoS One 2012; 7:e28006.
11. Yu ML, Chuang WL, Dai CY, et al. Different viral kinetics between hepatitis C virus genotype 1 and 2 as on-treatment predictors of response to a 24-week course of high-dose interferon-alpha plus ribavirin combination therapy. Transl Res 2006; 148:120-127.
12. Kamal SM, El Kamary SS, Shardell MD, et al. Pegylated interferon alpha-2b plus ribavirin in patients with genotype 4 chronic hepatitis C: The role of rapid and early virologic response. Hepatology 2007; 46:1732-1740.
13. Yu ML, Dai CY, Huang JF, et al. A randomised study of peginterferon and ribavirin for 16 versus 24 weeks in patients with genotype 2 chronic hepatitis C. Gut 2007; 56:553-559.
14. Von Wagner M, Huber M, Berg T, et al. Peginterferon-alpha-2a (40KD) and ribavirin for 16 or 24 weeks in patients with genotype 2 or 3 chronic hepatitis C. Gastroenterology 2005; 129:522-527
15. Tsang OT, Zee JS, Chan JM, et al. Chronic hepatitis C genotype 6 responds better to pegylated interferon and ribavirin combination therapy than genotype 1. J Gastroenterol Hepatol. 2010; 25:766-771.
16. Ferenci P, Laferl H, Scherzer TM, et al. Peginterferon alfa-2a and ribavirin for 24 weeks in hepatitis C type 1 and 4 patients with rapid virological response. Gastroenterology 2008; 135:451-458
17. Zeuzem S, Buti M, Ferenci P, et al. Efficacy of 24 weeks treatment with peginterferon alfa-2b plus ribavirin in patients with chronic hepatitis C infected with genotype 1 and low pretreatment viremia. J Hepatol 2006; 44:97-103.
