# Supplementary material for: 24 versus 48 Weeks of Peginterferon Plus Ribavirin in Hepatitis C Virus Genotype 6 Chronically Infected Patients with a Rapid Virological Response: A Non-Inferiority Randomized Controlled Trial
Source: PLoS One. 2015 Oct 28;10(10):e0140853. doi: 10.1371/journal.pone.0140853 (PMC4624894; doi:10.1371/journal.pone.0140853)
Supplement: S2 Protocol — (DOC) [file pone.0140853.s005.doc]

项目编号：2010011

**中山大学临床医学研究5010计划项目**

**任务书**

| 项目名称: | 基因6型慢性丙型肝炎的个体化优化治疗及长期预后的前瞻性研究 |
| --- | --- |
| 承担单位: | 中山大学附属第三医院 |
| 项目负责人: | 赵志新 |
| 联系电话:  手 机: | 020-85252378 |
|  | 13527873716 |
| 电子邮箱: | [Cqx200000@163.com](mailto:Cqx200000@163.com) |
| 起止时间: | 2011 年01月——2020年12月 |

二○一○年十一月制

**填 写 说 明**

1. 本任务书仅适用于我校满足相关条件的在编在岗教师填报项目立项、实施及验收的基本依据。
2. 填写任务书前，请先查阅《[中山大学临床医学研究5010计划管理办法（试行）](http://mso.sysu.edu.cn/column/ShowArticle.asp?ArticleID=238)》及《中[中山大学临床医学研究5010计划纲要](http://mso.sysu.edu.cn/column/ShowArticle.asp?ArticleID=237)》。
3. 本《任务书》各方可根据项目具体情况，就本《任务书》的未尽事宜协商签订附加条款。附加条款与本《任务书》正文具有同等法律效力。
4. 本《任务书》中的表格如位置不够请自行加页。
5. 《任务书》由申请人填写，经院系签署意见后，一式三份送医学科学处。

一、项目基本信息

| 项目名称 | | 基因6型慢性丙型肝炎的个体化优化治疗  及长期预后的前瞻性研究 | | | | | | |
| --- | --- | --- | --- | --- | --- | --- | --- | --- |
| 项目承担单位 | | 中山大学附属第三医院感染科 | | | | | | |
| 合作单 位 | 序号 | 单 位 名 称 | | | | | | |
|  | 1 | 广州市第八人民医院 | | | | | | |
|  | 2 | 中山市第二人民医院 | | | | | | |
|  | 3 | 番禺人民医院 | | | | | | |
|  | 4 | 中山大学公共卫生学院 | | | | | | |
|  | 5 |  | | | | | | |
|  | 6 |  | | | | | | |
| 项目负责人 | 姓 名 | 赵志新 | | | 性 别 | | 女 | |
|  | 出生年月 | 1962.02 | | | 学历 | | 博士研究生 | |
|  | 学 位 | 博士 | | | 专业 | | 内科传染病学 | |
|  | 职务 | 副主任 | | | 职称 | | 教授 | |
|  | 联系电话 | 13527873716 | | | E-mail | | Cqx200000@163.com | |
| 主要参加人员 | 姓名 | 性别 | 年龄 | 职称 | 学历 | 单位 | | 签名 |
|  | 蔡庆贤 | 男 | 29 | 主治  医师 | 硕士  研究生 | 中山大学  附属第三医院 | |  |
|  | 高志良 | 男 | 48 | 教授 | 博士  研究生 | 中山大学  附属第三医院 | |  |
|  | 崇雨田 | 男 | 48 | 教授 | 博士  研究生 | 中山大学  附属第三医院 | |  |
|  | 张晓红 | 女 | 44 | 副教授 | 博士  研究生 | 中山大学  附属第三医院 | |  |
|  | 林潮双 | 女 | 40 | 副教授 | 博士  研究生 | 中山大学  附属第三医院 | |  |
|  | 邓洪 | 男 | 38 | 副教授 | 博士  研究生 | 中山大学  附属第三医院 | |  |
|  | 许敏 | 女 | 49 | 主任  医师 | 本科 | 广州市  第八人民医院 | |  |
|  | 卫敏 | 女 | 49 | 主任  医师 | 本科 | 中山市  第二人民医院 | |  |
|  | 黄明寿 | 男 | 51 | 副主任  医师 | 本科 | 番禺人民医院 | |  |
|  | 任泽舫 | 男 | 47 | 副教授 | 博士  研究生 | 中山大学  公共卫生学院 | |  |
|  | 张英 | 女 | 36 | 主治  医师 | 硕士  研究生 | 中山大学  附属第三医院 | |  |
|  | 吴元凯 | 男 | 29 | 住院  医师 | 硕士  研究生 | 中山大学  附属第三医院 | |  |
|  | 许镇 | 男 | 29 | 住院  医师 | 本科 | 中山大学  附属第三医院 | |  |
|  | 邵晓琼 | 女 | 24 | 硕士  研究生 | 本科 | 中山大学  附属第三医院 | |  |
|  | 朱瑞华 | 女 | 24 | 办事员 | 本科 | 中山大学  附属第三医院 | |  |
|  | 卢翠容 | 女 | 45 | 主管  护师 | 大专 | 中山大学  附属第三医院 | |  |
|  | 洪春霞 | 女 | 33 | 主管  护师 | 大专 | 中山大学  附属第三医院 | |  |
|  | 胡朝霞 | 女 | 28 | 技师 | 本科 | 中山大学  附属第三医院 | |  |

| 项目主要研究内容的国内外现状和发展趋势，包含以下三个方面：  **1、项目内容摘要**  慢性丙型肝炎（Chronic hepatitis C，CHC）严重危害人类的健康。长效干扰素联合利巴韦林是目前公认的治疗CHC最有效的方案，然而高昂的治疗费用与长期治疗导致的各种不良反应严重的阻碍抗病毒治疗的开展。个体化优化治疗是根据患者的个体特征来定制治疗方案，旨在提高患者的持续病毒学应答率，并减少不必要的治疗。目前关于个体化优化治疗的研究已在基因1、2、3、4型CHC上取得了令人满意的进展。基因6型CHC因地理分布上的局限性，其个体化治疗的研究严重落后。广东省是CHC高发的省份，研究证实，基因6型CHC在广东地区的流行呈上升趋势。然而目前对基因6型CHC的治疗只能沿用常规的48周疗程方案，这不仅造成医疗资源的浪费，也增加了各种不良反应发生的风险，更不利于持续病毒学应答率的提高。课题组拟通过多中心、前瞻性、开放性、随机对照试验，以快速病毒学应答及早期病毒学应答等指标为依据，探索基因6型CHC的个体化优化治疗方案。本研究借助本地区CHC基因型分布结构的特殊性，强调以人为本的精神，首次提出对基因6型CHC治疗方案进行个体化优化，研究结果将对临床治疗基因6型CHC具有重要的指导意义。  **2、项目的意义和必要性**  丙型肝炎病毒(Hepatitis C Virus，HCV)慢性感染可导致肝脏慢性炎症坏死和纤维化，部分患者可发展为肝硬化甚至肝细胞癌(Hepatocellular carcinoma, HCC)，是引起终末期肝病的最主要原因之一。据世界卫生组织统计，全球HCV的感染率约为3％，估计约1.7亿人感染了HCV，每年新发丙型肝炎病例约3.5万例^[1]^。我国是HCV流行最严重的国家之一，1992-1995年全国第二次肝炎流调显示，我国HCV感染率为0.9-5.1%,平均3.2%。  HCV分为6个主要基因型和80多个基因亚型。其中1-3型呈世界性流行，而4-6型呈地域性分布。其中基因4型主要分布在埃及、中东和非洲中部，基因5型主要在非洲南部，而基因6型主要分布在东南亚的越南、香港、澳门等地区^[2]^。  既往的研究显示，我国HCV流行以1b和2a基因型较为常见，并以1b型为主；某些地区有1a、2b和3b型。6型主要见于香港、澳门等地区^[3]^。然而，近来的研究提示国内基因6型CHC流行率呈上升趋势^[4,5]^。  吕凌等^[6]^ 2002年对国内献血员中HCV感染者的基因分型统计结果显示，国内1b、2a、6a基因型所占的比例依次为66.2%、13.7%、10.1%，而在广东地区6a型占21.2%（14/66），超过2a型的比例。2010年再次的统计结果显示，6a型在广东地区所占的比例上升到49.7%，超越了1b型，而国内其他省份的6a型的比例并无太多变化，为 10.6%^[7]^。近期课题组通过对HCV的CORE及NS5b两个片段进行测序并建基因树的方法，对广东地区慢性丙型肝炎（Chronic Hepatitis C，CHC）患者的HCV进行基因分型。结果发现，1b型所占比例最高，占50%（112/224），其次是6a型，占34%（78/224），2a和3b所占比例相对较少，分别为11%（26/224）与5%（12/224）（结果未发表）。虽然CHC患者与献血员中HCV感染者病毒基因型的分布结构存在一定的差异，但上述结果均提示6a型流行的严重性。  关于基因6型CHC的治疗及临床预后, 尚缺少相关的临床研究。 |
| --- |

二、主要研究内容

| **3、项目的研究内容、国内外现状和发展趋势**  目前临床上主要采用干扰素联合利巴韦林治疗CHC，并以治疗结束后随访半年时的HCVRNA转阴，即持续病毒学应答（[Sustained Virologic Response](http://hepatitis.about.com/od/treatment/f/SVR.htm)，SVR）作为疗效的判断指标。聚乙二醇干扰素因其半衰期长，给药方便，SVR率更高等优点，目前被各个版本指南推荐为首选药物。临床研究证实，聚乙二醇干扰素α2a联合利巴韦林治疗CHC48周的SVR率可达到56%，显著高于普通干扰素联合利巴韦林（44%，P<0.001）或单独使用聚乙二醇干扰素α2a（29%, P<0.001）治疗48周的SVR率^[8]^。干扰素在发挥抗病毒作用的同时，也产生一定的副作用。临床常见副作用包括：流感样症状、骨髓抑制、抑郁、诱发糖尿病、甲亢、自身免疫性疾病等。不良反应的发生率随着疗程的延长而递增。为了提高SVR，减少不必要的治疗，各地临床专家开始探索个体化优化治疗方案。已知宿主与病毒多个方面的因素影响干扰素治疗CHC的SVR率。其中病毒基因型、快速病毒学应答（Rapid viralogic Response，RVR；疗程第4周时HCVRNA转阴）、早期病毒学应答（Early viralogic Response，EVR；疗程第12周时HCVRNA转阴）是目前已知的影响SVR最重要的因素^[9]^。基因型作为最重要的基线因素对干扰素联合利巴韦林的疗程起到决定性的作用。而治疗过程中的RVR对SVR有较好的阳性预测价值，EVR则有较好的阴性预测价值，这两个时间点的应答情况是目前个体化优化治疗的重要依据。  近年来，基于病毒基因型、RVR及EVR而设计的一些优化治疗的临床研究取得了令人鼓舞的结果。  [Hadziyannis SJ](http://www.ncbi.nlm.nih.gov/pubmed?term="Hadziyannis SJ"%5BAuthor%5D)等^[10]^分别比较基因1型和2、3型慢性HCV感染使用长效干扰素联合利巴韦林治疗24周和48周的疗效，结果发现，对于基因1型，48周疗程的SVR率显著高于24周疗程。对于基因2、3型，24周和48周疗程的疗效无显著性差异。因此目前常规对基因1型患者常规采用48周疗程治疗，对2、3型患者常规采用24周疗程治疗。  而Jensen DM等^[11]^研究发现，达到RVR的基因1型慢丙肝患者接受24周治疗，SVR率可以达到89%。Moreno C等^[12]^的研究进一步证实，基因1型患者如果满足：基线病毒量<400,000 IU/ml，治疗4周时HCVRNA转阴，则治疗24周与48周的SVR无显著差异。  von Wagner M等^[13]^将达到RVR的基因2、3型HCV患者随机分配到16周与24周治疗组接受长效干扰素联合利巴韦林治疗，结果显示，两组SVR率无显著差异（82% VS 80%）。  Pearlman BL等^[14]^将治疗12周时HCVRNA仍未转阴的基因1型CHC患者随机分配到48周和72周疗程组，结果发现72周疗程的SVR率显著高于48周疗程（38% VS 18%）。  而目前关于长效干扰素联合利巴韦林治疗基因6型CHC的临床经验较少。仅有一项前瞻性的研究结果发表，这项在中国香港进行的配对研究比较了70例基因1型和70例基因6型CHC患者对48周疗程PEG IFN + RBV的疗效，结果发现基因6型CHC在早期应答，治疗结束时应答及随访终点应答均优于基因1型患者(81.4% vs 60% P = 0.005；88.6% vs 74.3%, P = 0.03；75.7% vs 57.1%, P = 0.02)^[15]^。该研究提示基因6型CHC对长效干扰素联合利巴韦林治疗的应答优于基因1型CHC患者。然而，如何对基因6型CHC进行个体化的治疗，目前尚缺乏可靠的循证医学证据，而各地指南对于基因6型CHC的具体疗程也无明确的推荐。目前国内对基因6型CHC的治疗均沿用基因1型的48周疗程，这样不仅造成医疗资源的浪费，增加不良反应的风险，也不利于提高SVR率。因此，如何对基因6型CHC患者进行个体化优化治疗已成为目前临床上迫切需要解决的问题。  课题组拟通过多中心、前瞻性、开放性、随机对照试验，以RVR及早期病毒学应答（即治疗12周时检测HCV RNA转阴，Early viralogic Response，RVR）等指标为依据，探索基因6型CHC患者的个体化优化治疗及长期预后。为临床个体化治疗基因6型CHC患者提供必要的循证医学证据。 |
| --- |
| 参考文献：  [1] www.who.int/immunization/topics/hepatitis_c/en/ [2][Antaki N](http://www.ncbi.nlm.nih.gov/pubmed?term="Antaki N"%5BAuthor%5D), [Craxi A](http://www.ncbi.nlm.nih.gov/pubmed?term="Craxi A"%5BAuthor%5D), [Kamal S](http://www.ncbi.nlm.nih.gov/pubmed?term="Kamal S"%5BAuthor%5D), et al. The neglected hepatitis C virus genotypes 4, 5 and 6: an international consensus report. [Liver Int.](javascript:AL_get(this, 'jour', 'Liver Int.');) 2010, 30(3):342-55  [3]丙型肝炎防治指南.中华传染病杂志，2004，22（2）：131～136.  [4] YouQian Zhou, XiaoHong Wang, Qing Mao,et al. Changes in modes of hepatitis C infection acquisition and genotypes in southwest China. Journal of Clinical Virology, 2009(46):230–233  [5][Vutien P](http://www.ncbi.nlm.nih.gov/pubmed?term="Vutien P"%5BAuthor%5D), [Nguyen NH](http://www.ncbi.nlm.nih.gov/pubmed?term="Nguyen NH"%5BAuthor%5D), [Trinh HN](http://www.ncbi.nlm.nih.gov/pubmed?term="Trinh HN"%5BAuthor%5D),et al. Similar treatment response to peginterferon and ribavirin in Asian and Caucasian patients with chronic hepatitis C. [Am J Gastroenterol.](javascript:AL_get(this, 'jour', 'Am J Gastroenterol.');)2010,105(5):1110-1115  [6]Lu L, Nakano T, He Y,et al. Hepatitis C virus genotype distribution in China: predominance of closely related subtype 1b isolates and existence of new genotype 6 variants. J Med Virol 2005,75:538-549.  [7][Fu Y](http://www.ncbi.nlm.nih.gov/pubmed?term="Fu Y"%5BAuthor%5D), [Wang Y](http://www.ncbi.nlm.nih.gov/pubmed?term="Wang Y"%5BAuthor%5D), [Lu L](http://www.ncbi.nlm.nih.gov/pubmed?term="Lu L"%5BAuthor%5D),et al.New trends of HCV infection in China revealed by genetic analysis of viral sequences determined from first-time volunteer blood donors. [J Viral Hepat.](javascript:AL_get(this, 'jour', 'J Viral Hepat.');) 2010 Feb 25.[Epub ahead of print]  [8]FriedMW, ShiffmanML, Reddy KR, et al. Peginterferon alfa-2a plus ribavirin for chronic hepatitis C virus infection. N Engl J Med 2002,347:975-982  [9]Yu JW, Wang GQ, Sun LJ,et al.Predictive value of rapid virological response and early virological response on sustained virological response in HCV patients treated with pegylated interferon alpha-2a and ribavirin. J Gastroenterol Hepatol 2007,22:832-836  [10][Hadziyannis SJ](http://www.ncbi.nlm.nih.gov/pubmed?term="Hadziyannis SJ"%5BAuthor%5D), [Sette H Jr](http://www.ncbi.nlm.nih.gov/pubmed?term="Sette H Jr"%5BAuthor%5D), [Morgan TR](http://www.ncbi.nlm.nih.gov/pubmed?term="Morgan TR"%5BAuthor%5D),et al. Peginterferon-alpha2a and ribavirin combination therapy in chronic hepatitis C: a randomized study of treatment duration and ribavirin dose. [Ann Intern Med.](javascript:AL_get(this, 'jour', 'Ann Intern Med.');) 2004,140(5):346-55.  [11]Jensen DM, Morgan TR, Marcellin P,et al. Early identiﬁcation of HCV genotype 1 patients respond-ing to 24 weeks peginterferon alpha-2a (40 kd)/ribavirin therapy. HEPATOLOGY 2006,43:954-960  [12][Moreno C](http://www.ncbi.nlm.nih.gov/pubmed?term="Moreno C"%5BAuthor%5D), [Deltenre P](http://www.ncbi.nlm.nih.gov/pubmed?term="Deltenre P"%5BAuthor%5D), [Pawlotsky JM](http://www.ncbi.nlm.nih.gov/pubmed?term="Pawlotsky JM"%5BAuthor%5D),et al.Shortened treatment duration in treatment-naive genotype 1 HCV patients with rapid virological response: a meta-analysis. [J Hepatol.](javascript:AL_get(this, 'jour', 'J Hepatol.');)2010,52(1):25-31  [13]von Wagner M, Huber M, Berg T,et al. Peginterferon-alpha-2a (40KD) and ribavirin for 16 or 24 weeks in patients with genotype 2 or 3 chronic hepatitis C. Gastroenterology 2005,129:522-527.  [14] Pearlman BL, Ehleben C, Saifee S. Treatment extension to 72 weeks of peginterferon and ribavirin in hepatitis c genotype1 infected slow responders. HEPATOLOGY 2007,46:1688-1694.  [15] Tsang, Owen T-Y, Zee, et al. Chronic hepatitis C genotype 6 responds better to pegylated interferon and ribavirin combination therapy than genotype 1. [Journal of Gastroenterology and Hepatology](http://www.ingentaconnect.com/content/bsc/jgh;jsessionid=337rk518iegke.victoria), 2010, 25(6):766-771 |

三、项目拟采取的研究方法及技术路线（或实施方案）

| **1.本研究为前瞻性、多中心、开放性、随机对照试验。**  **2.病人的来源：**研究对象为基因6型CHC患者，来自本课题协作组的四个临床中心：中山大学第三医院、广州市第八人民医院、广州市番禺人民医院、中山市第二人民医院等。  **3.入选标准**   - 年龄18到70岁之间（含18和70岁），性别不限； - 诊断符合2004年《慢性丙型肝炎防治指南》关于CHC的诊断标准, 丙肝抗体阳性且HCVRNA阳性，并持续超过6个月以上； - 经测序建树的方法确定基因型为6型； - 转氨酶升高达正常值上限1.5倍以上； - 代偿期肝病；总胆红素低于2 mg/dL;白蛋白大于36 g/L; 凝血酶原活动度80%; 无腹水，肝性脑病或消化道出血）.   **4.排除标准：**   - 肝硬度测定大于12.5 KPa； - CT或者MR证实肝癌； - 合并HBV，HIV感染者； - 在入选期病人白细胞<3000 cells/mm3，中性粒细胞计数<1500 cells/mm3或血小板计数 <90,000 cells/mm3，入选期女性病人血红蛋白< 12 g/dL 或男性病人血红蛋白<13 g/dL - 接受抗病毒，抗肿瘤或免疫抑制剂治疗者 - 孕妇（治疗开始前妊娠反应阳性）或哺乳期妇女 - 其他慢性肝病（如自身免疫性肝炎）病史或临床症状 - 严重精神疾病史，特别是抑郁症史；免疫性疾病史; 慢性肺病伴肺功能障碍史；严重心血管疾病史；甲状腺疾病未被有效治疗控制史 - 其他原因研究者认为不适宜参加试验   **5. 剔除病例标准（**已入组病例但符合以下之一者，应予剔除**）**   - 误诊； - 符合排除标准； - 未曾用药者； - 无任何检测记录者； - 由于使用某种禁用的药物，以致无法评价药效。 - 剔除的病例应说明原因，其CRF表应保留备查。不作疗效统计分析，但至少接受一次治疗，且有记录者，可参加不良反应分析。   **6. 退出（脱落）病例标准**   - 因以下原因未完成全部临床研究的入组病例应视为脱落： - 病人自行退出（疗效太差，不良反应等）； - 失访； - 研究者原因的退出（依从性差；出现严重的合并症和并发症专家组认为需要退出；尿妊娠试验阳性；严重不良事件）； - 虽然完成试验，但服药量不在应服量的80%-120%范围内。 - 专家组认为需要退出的其他情况。 - 所有脱落的病例应详细记录原因，并留取终点标本，将其最后一次的主要疗效检测结果转接为最终结果进行统计分析，其CRF表应保留备查。   **7．病例分组：**  符合入选标准的患者需先进行PEG-IFN 180µg+ RBV10.6-15mg/d/kg 4周的治疗后再进行RVR评价，获得RVR者通过随机分配进入A组或B组。未获得RVR者继续治疗，第12周时进行EVR评价，获得EVR者继续进行标准治疗至48周，未获得EVR者通过随机分配进入C组或D组。治疗期间受试者因不良反应等原因更换过治疗方案或因其他原因脱落，则不再进行分组和随机，根据更改后的方案继续进行治疗和随访。（随机方法见附件一）  A组：达到RVR的患者，随机分配入24周疗程（优化方案1）；  B组：达到RVR的患者，随机分配入48周疗程（标准方案）；  A与B组比较：可以得出对于达到RVR的基因6型CHC患者能否可将疗程缩短至24周；  **8. 样本量**  本研究A、B组为非劣性试验，根据以往经验及统计学的一般要求，取α=0.05,β=0.2,等效标准δ=0.15,48疗程对达到RVR的CHC的疗效为P=0.85，根据公式N= 2×(Uα+Uβ)2×P(1-P) /δ2，N=69，假定10%的脱落率，则A、B两组共需要152例RVR的基因6型CHC患者。  **9.治疗方案：**  **标准治疗：**Peg-IFNα-2a联合RBV，Peg-IFNα-2a：180μg, 皮下注射，1次/周，RBV：10.6-15mg/kg/day,口服，疗程48周；  **优化方案1：**Peg-IFNα-2a联合RBV， Peg-IFNα-2a：180μg, 皮下注射，1次/周，RBV：10.6-15mg/kg/day,口服，疗程24周；  **个体化治疗方案：**治疗过程中，若患者因各种原因（白细胞、血小板降低，甲状腺功能异常等）不能耐受上述治疗，可酌情减少剂量（包括干扰素和利巴韦林）。  **10.观察指标**   - 人口学资料：性别，年龄，民族，职业等； - 体检：体温、心率、呼吸、血压、身高、体重、及体格检查等； - 实验室及辅助检查：   所有受试者筛选期（入选前两周内）进行：尿妊娠实验、血常规、尿常规、生化（肝肾功能、血糖、血脂、电解质）、PT/APTT/PTA/INR、甲胎蛋白（AFP）、乙肝五项、HIV抗体、HCV抗体、HCV基因分型、HCV-RNA定量、腹部B超、胸片、心电图、自身抗体（ANA、AMA、SMA）、甲状腺功能（T3、T4、FT3、FT4、TSH）、眼底检查、肝脏活检。按照《血清的留取、处理、保存、运送与接收标准操作规程》的要求分离和保存血清标本，待中心实验室统一检测。  治疗后A/B：于随访1（入选后2W）留取血清标本（同基线）。  治疗后A/B组：于随访2（入选后4W）、随访3（入选后12W）进行HCV-RNA定量的检测。  治疗后A/B组：于随访4（入选后24W）进行尿常规、PT/APTT/PTA/INR、甲胎蛋白（AFP）、HCV-RNA定量、腹部B超、自身抗体（ANA、AMA、SMA）、甲状腺功能（T3、T4、FT3、FT4、TSH）的检测。  治疗后A/B组：于随访5（入选后48W）、随访6（入选后72W）、随访7（入选后96W）进行甲胎蛋白（AFP）、HCV-RNA定量、腹部B超、自身抗体（ANA、AMA、SMA）、甲状腺  功能（T3、T4、FT3、FT4、TSH）的检测。随访5作为优化方案1治疗的A组受试者的末次访视，随访6作为标准方案治疗的B、C组受试者的末次访视，随访7作为使用优化方案2治疗的D组受试者的末次访视，还需加进行尿妊娠实验和眼底检查，并进行肝脏活检和标本的制备保存（方法同基线）。  每次随访均需留取中心实验室检测所需血清标本（方法同基线）。  除随访1以外每次随访均需进行血常规、血生化（肝肾功能、血糖、血脂）的检测。   - 合并用药记录； - 不良事件记录； - 依从性评价：依从性（%）=（已服药量/应服药量）×100%  10. 主要检测指标：  - **HCV RNA定量：**统一使用Roche公司的COBAS Taqman定量检测试剂盒，对0、4周、12周、24周、48周、治疗终点、停药24周血清标本进行检测，HCV RNA<50IU/ml为阴性；HCV RNA≥50IU/ml为阳性。 - **HCV基因分型：**根据对HCV病毒CORE片段的测序结果，建基因树进行分型。 - **肝组织病理检查：**按2000年西安会议提出的标准分级分期，慢性肝炎炎症分级标准有4级，纤维化分期标准有4期。同时按照修正的Knodell肝组织炎症活动度(histological activity index, HAI)评分，为0～18分，纤维化评分为0～6分。  11. 疗效评价：  - **快速病毒学应答（Rapid Virologic Response， RVR）:** 治疗4周，HCV RNA< 50IU/ml。 - **早期病毒学应答（early virus response, EVR）：**治疗12周，HCV RNA<50IU/ml（完全早期病毒学应答，complete EVR，cEVR），或HCV RNA值与基线比较下降≥2Log（部分早期病毒学应答，partial EVR，pEVR）。 - **治疗结束时病毒学应答（end treated virus response, ETVR）：**治疗结束时，HCV RNA<50IU/ml。 - **持续病毒学应答（sustained virus response, SVR）：**治疗结束，停药24周随访时，HCV RNA<50IU/ml。 - **无应答（NVR）：**指从未获得EVR、ETVR及SVR者。 - **复发（relapse）：**指治疗结束时为HCV RNA<50IU/ml，但停药后HCV RNA又变为阳性。 - **治疗中反弹（break through）：**治疗期间曾有HCV RNA载量降低或阴转，但尚未停药即出现HCV RNA载量上升或阳转。  13. 终点指标  - **主要终点指标：**以SVR率为指标评价不同组别的疗效。 - **次要终点指标：**不同组别的药物安全性及耐受性，包括不良反应发生率，退出率。  14. 研究技术路线 治疗药物：派罗欣 180µg/w+ RBV10.6-15mg/d  随机分组  1：1  **A组: 派罗欣 180µg/w+ RBV**  **10.6-15mg/d（24周疗程）**  **随访24周**  **获得RVR的基因6型慢性丙肝152例**  **B组: 派罗欣 180µg/w+ RBV**  **10.6-15mg/d（48周疗程）**  **随访24周** 15.知情同意 每一位患者入选本研究前，研究医师有责任以书面文字形式，向其或其指定代表完整、全面地介绍本研究的目的、性质、程序和可能的受益及风险。应让患者知道他们有权随时退出本项研究。入选前必须给每位患者一份书面的知情同意书（以附录形式包括于方案之中）使受试者了解后表示同意。并自愿签署知情同意书后，方可入选进行临床试验。知情同意书应作为临床试验的原始资料之一，保存备查。为保护受试者隐私，病例报告表上不应出现受试者的姓名。研究者应按受试者的代码确认其身份并记录。  **16．统计分析**  人口统计资料中的连续变量应计出平均值，中间值，标准差，最大和最小值；分类资料在统计表中列出频数和百分数。主要和次要终点的反应率使用百分比和95％的可信区间计算，成对t检验用于建议连续性变量。 |
| --- |

四、研究目标和考核指标

| **（1）2011年（准备阶段）**：  **年度任务**  ① 制定基因6型CHC个体化优化治疗研究的具体操作规程；  ② 完成clinical trial的临床试验登记；  ③ 建立稳定的基因分型方法；  ④ 开始入组病人进行治疗。  **年度考核指标**：  ① 完成基因6型CHC相关CRF表的设计和印制；  ② 参加单位的研究人员启动会和培训会；  ③ 分配参加研究人员的具体工作任务；  ④ 请有关统计专家设定好随机号并分配给各研究中心；  ⑤ 建立项目随访资料库、血清库、病理库；  **（2）2012年（实施阶段）**：  **年度任务**  ① 召开参加单位的研究人员总结会，公开项目实施情况，就第一年项目实施遇到的问题进行讨论解决；  ② 继续入组病人；  ③ 收集并保存相应时间内的系列血清、血浆、PBMC、血块、肝组织样本；  ④ 检测病人基因组的SNP，分析其与早期应答的相关性；  **年度考核指标**：  ① 入组病例达到152人以上；  ② 完成相应时间内的系列血清、血浆、PBMC、血块、肝组织样本的收集和保存；  **（3）2013年（实施阶段）**：  **年度任务**  ① 召开参加单位的研究人员总结会，就前两年项目实施情况进行总结，分配年度任务；  ② 继续入组病人；  ③ 收集并保存相应时间内的系列血清、血浆、PBMC、血块、肝组织样本；  ④ 进行资料的收集、整理、分析及论文的撰写。  **年度考核指标**：  ① 完成A、B两组的入组、治疗、治疗结束后随访24周以上；  ② 完成收集相应时间内的系列血清、血浆、PBMC、血块、肝组织样本，并建库保存；  ③ 完成SCI论文2篇：一篇“比较24周与48周疗程长效干扰素联合利巴韦林治疗达到RVR的基因6型慢性丙型肝炎的疗效与安全性” （目标，IF值10.0以上英文期刊）；一篇就“长效干扰素联合利巴韦林治疗基因6型慢性丙型肝炎的疗效预测指标”发表SCI论文（目标IF值5.0左右的英文期刊）。  **（4）2014-2016年（实施阶段）**：  ① 完成C、D两组的入组、治疗、治疗结束后随访24周以上；  ② 进一步丰富样本库；  ③ 完成相应时间内新收集样本的相关检测；  ④ 发表SCI论文1篇：一篇“比较48周与72周疗程长效干扰素联合利巴韦林治疗未达到EVR的基因6型慢性丙型肝炎的疗效与安全性”（IF值5.0以上）。  **（5）2017-2020年（随访及方案推广阶段）**：  ① 对完成上述治疗的患者进行长期随访；  ② 以特定范围作为研究对象，对个体化优化治疗方案进行推广，鼓励患者积极配合治疗；  ③ 完成收集相应时间内的系列血清、血浆、PBMC、血块、肝组织样本，入库保存；  ④ 发表SCI论文3篇：a.在各组患者治疗与长期临床转归（死亡率、HCC及LC发生率）及影响因素方面发表1篇（IF值5.0以上）；b.在药物经济学方面发表1篇（IF值3.0左右）；c.在病毒学、组织学疗效与长期临床转归的关系方面发表1篇（IF值3.0左右）；  ⑤编写相关专著1部；  ⑥成为全国性继续教育项目，并主办3-5期学习班；  ⑦力争获得省级科技进步一等奖；国家科技进步二等奖。 |
| --- |

五、项目承担单位提供的技术及条件保障

| （包括临床研究中心建设情况以及提供的人、财、物等支撑条件，该栏目由承担单位负责填写）  项目依托单位为中山大学附属第三医院传染科，属广东省重点学科，广东省病毒性肝炎临床与实验研究中心及校“211”工程重点建设学科，卫生部临床药理基地及广东省病毒性肝炎临床与实验研究中心，具有雄厚的临床实力，历史悠久，资源丰富，收治肝病患者的床位数达200张以上，门诊量14万人次/年，是广东省病毒性肝炎患者来源最多的专科，具备丰富的丙肝治疗经验，有完善的病人随访系统。已建立起丙肝专家门诊以统一接诊慢性丙型肝炎患者，建立起随访门诊系统、丙肝患者病情档案和“三库（血清库、病理库和数据库）”等临床研究平台。  实验室有专职研究人员及技术人员15名，有核酸研究相应的荧光定量PCR仪（Taqman5700）、凝胶分析系统和电泳及转印设备，并有中心试验室的资源可供利用，中心试验室有荧光-发光检测仪、 流式细胞仪（FACS，BD公司）、高压液相系统、DNA序列分析仪（ABI 3100-Avant）和超速离心机等设备，足够完成本项目的研究。  单位负责人（签章） 单位（公章） 年 月 日 |
| --- |

**六、项目经费支出总预算**

单位：万元（保留两位小数）

| **科目名称** | | | | **合计** | **学校**  **经费** | **单位**  **配套** | **自筹经费** |
| --- | --- | --- | --- | --- | --- | --- | --- |
| **合 计** | | | | 200.00 | 100.00 | 100.00 | 0 |
| 1. 科研业务费 | | | | 138.00 | 40.5 | 97.50 | 0 |
| （1）材料费 | | | | 118.00 | 20.50 | 97.50 | 0 |
| （2）差旅费 | | | | 5.00 | 5.0 | 0 | 0 |
| （3）会议费 | | | | 10.00 | 10.00 | 0 | 0 |
| （4）出版物/文献/信息传播/知识产权事务费 | | | | 5.00 | 5.00 | 0 | 0 |
| （5）统计分析费 | | | | 0 | 0 | 0 | 0 |
| 2. 合作费 | | | | 38.50 | 38.50 | 0 | 0 |
| 3. 国际合作与交流费 | | | | 10.00 | 10.00 | 0 | 0 |
| 4. 临床病例观察随访费 | | | | 0 | 0 | 0 | 0 |
| 5. 专家咨询费 | | | | 5.00 | 5.00 | 0 | 0 |
| 6. 人员劳务费 | | | | 6.00 | 6.00 | 0 | 0 |
| 7. 管理费 | | | | 2.50 | 0 | 2.50 | 0 |
| 8．其它 | | | | 0 | 0 | 0 | 0 |
| **年度预算** | 2011年 | 2012年 | 2013年 | 2014年 | 2015年 | 2016年 | …… |
|  | 20.00万元 | 20.00万元 | 20.00万元 | 20.00万元 | 20.00万元 | 20.00万元 | …… |
| 对经费预算的其他说明: 本单位配套资金需按2.5%的比例缴纳管理费。 | | | | | | | |

**关于“项目经费支出总预算”中“合作费”支出的说明：**

1. 合作费中的2.50万元根据支付给中山大学公共卫生学院作为“专家咨询费支出”。
2. 36.00万作为其他三家合作医院的研究费。本项目采用竞争入组，研究中心每完成一例，承担单位将给与研究中心2500元（其中专家咨询费500元/例，人员劳务费2000元/例）的研究经费。具体支出预算如下：

单位：万元（保留两位小数）

| **科目名称** | | | | **合计** | **广州市第八人民医院** | **中山市第二人民医院** | **番禺人民医院** |
| --- | --- | --- | --- | --- | --- | --- | --- |
| **合 计** | | | | 36.00 | —— | —— | —— |
| 1. 科研业务费 | | | | 6.00 | 2.00 | 2.00 | 2.00 |
| （1）会议费 | | | | 3.00 | 1.00 | 1.00 | 1.00 |
| （2）出版物/文献/信息传播/知识产权事务费 | | | | 3.00 | 1.00 | 1.00 | 1.00 |
| 2. 专家咨询费 | | | | 6.00 | 0.05/例 | 0.05/例 | 0.05/例 |
| 3. 人员劳务费 | | | | 24.00 | 0.20/例 | 0.20/例 | 0.20/例 |
| **年度预算** | 2011年 | 2012年 | 2013年 | 2014年 | 2015年 | 2016年 | …… |
|  | 7.50万元 | 5.00万元 | 5.00万元 | 5.00万元 | 5.00万元 | 5.00万元 | …… |
| 对经费预算的其他说明:第一年先拨给各个临床中心2.50万，以后根据实际完成的病例数调整。 | | | | | | | |

**2011年度项目合作费支出预算**

| **科目名称** | **合计** | **广州市第八人民医院** | **中山市第二人民医院** | **番禺人民医院** | **中山大学公共卫生学院** |
| --- | --- | --- | --- | --- | --- |
| **合 计** | 7.50 | 2.40 | 2.40 | 2.40 | 0.30 |
| 1. 科研业务费 | 1.20 | 0.40 | 0.40 | 0.40 | 0 |
| （1）会议费 | 0.60 | 0.20 | 0.20 | 0.20 | 0 |
| （2）出版物/文献/信息传播/知识产权事务费 | 0.60 | 0.20 | 0.20 | 0.20 | 0 |
| 2. 专家咨询费 | 1.50 | 0.40 | 0.40 | 0.40 | 0.30 |
| 3. 人员劳务费 | 4.80 | 1.60 | 1.60 | 1.60 | 0 |

**七、2011年度项目经费支出预算**

单位：万元（保留两位小数）

| **科目名称** | **合计** | **学校经费** | **单位配套** | **自筹经费** |
| --- | --- | --- | --- | --- |
| **合 计** | 20.00 | 10.00 | 10.00 | 0 |
| 1. 科研业务费 | 11.25 | 1.50 | 9.75 | 0 |
| （1）材料费 | 9.75 | 0 | 9.75 | 0 |
| （2）差旅费 | 0.50 | 0.50 | 0 | 0 |
| （3）会议费 | 1.00 | 1.00 | 0 | 0 |
| （4）出版物/文献/信息传播/知识产权事务费 | 0 | 0 | 0 | 0 |
| （5）统计分析费 | 0 | 0 | 0 | 0 |
| 2. 合作费 | 7.50 | 7.50 | 0 | 0 |
| 3. 国际合作与交流费 | 0 | 0 | 0 | 0 |
| 4. 临床病例观察随访费 | 0 | 0 | 0 | 0 |
| 5. 专家咨询费 | 0 | 0 | 0 | 0 |
| 6. 人员劳务费 | 1.00 | 1.00 | 0 | 0 |
| 7. 管理费 | 0.25 | 0 | 0.25 | 0 |
| 8．其它 | 0 | 0 | 0 | 0 |
| 对经费预算的其他说明: 第一年先拨给各个临床中心2.50万，以后根据实际完成的病例数及总经费预算做出调整。 | | | | |
