# Supplementary material for: 24 versus 48 Weeks of Peginterferon Plus Ribavirin in Hepatitis C Virus Genotype 6 Chronically Infected Patients with a Rapid Virological Response: A Non-Inferiority Randomized Controlled Trial
Source: PLoS One. 2015 Oct 28;10(10):e0140853. doi: 10.1371/journal.pone.0140853 (PMC4624894; doi:10.1371/journal.pone.0140853)
Supplement: S1 Text — (PDF) [file pone.0140853.s006.pdf]

The Translation of "Clinical Research Ethical Approval by the Ethics Committee of the Third Affiliated Hospital of Sun Yat-Sen University ([2010]2-53) "

Approved No. of ethic committee: 中大附三医伦 [2010]2-53

Project Title	The prospective study of individualized optimized treatment strategy of hepatitis C virus genotype 6 chronically infected patients		
Department	Department of Infectious Diseases,	Principle Investigator	Zhixin Zhao
Project source	Sun Yat-Sen University Clinical Research 5010 Program		
Financial sources	Sun Yat-Sen University Clinical Research 5010 Program		

Document checked

1. Trial protocol
2. informed consent

Decision

✓ Approved

☐ To revise and recheck

☐ Declined

January 7th, 2011

Notes

1. The duty, manning and working procedure of this Ethics Committee comply with the "ethics review method for ethical biomedical research involving human" published by Ministry of Health of the People's Republic of China.
2. Records should be filed to the Ethics Committee once the project was approved by the Sun Yat-Sen University Clinical Research 5010 Program.
3. Any revision involving the study protocol and informed consent could only be made after being approved by the Ethics Committee.
4. Any serious adverse event in the study should be reported to the Ethics Committee.
5. A summary report should be submitted to the Ethics Committee once the study was finished.

The Third affilicated Hospital of Sun Yat-sen University medical ethics committee
Address: 600 Tianhe road, Guangzhou Zip code: 510630 Tel: +862085253099

Translated by Qingxian Cai

Qing Xian Cai
